# Improving Access to Malaria Rapid Diagnostic Test in Niger State, Nigeria: An Assessment of Implementation up to 2013

**DOI:** 10.1155/2016/7436265

**Published:** 2016-03-06

**Authors:** Olatunji Joshua Awoleye, Chris Thron

**Affiliations:** ^1^OREEP NIG Ltd., State House, Block I, No. 6, Karu Site, Karu, AMAC, Abuja 900110, Nigeria; ^2^Department of Mathematics, Texas A&M University-Central Texas, 1001 Leadership Place, Killeen, TX 76549, USA

## Abstract

Nigeria's 2009–2013 malaria strategic plan adopted WHO diagnosis and treatment guidelines, which include the use of rapid diagnostic tests (RDTs) prior to prescribing treatment with artemisinin combination therapies (ACTs). The current study explores accessibility barriers to the use of RDTs in Niger State and makes recommendations for improving the uptake of RDTs. The study employs literature review, review of data from the Niger State Health Management Information System for January–October 2013, and application of Peters' conceptual framework for assessing access to health services. Data showed that 27 percent of public health facilities (HFs) implemented RDTs, with the aid of donor funds. In these facilities, 77 percent of fever cases presented during the study period were tested with RDTs; 53 percent of fever cases were confirmed cases of malaria, while 60 percent of fever cases were treated. Stockouts of RDTs were a major constraint, and severe fever tended to trigger presumptive treatment. We conclude that although implementation of RDTs led to a reduction in the use of ACTs at HFs, more substantial reduction could be achieved if the state government directed more resources towards the acquisition of RDTs as well as raising the level of awareness of potential users.

## 1. Introduction

**Figure 1:**
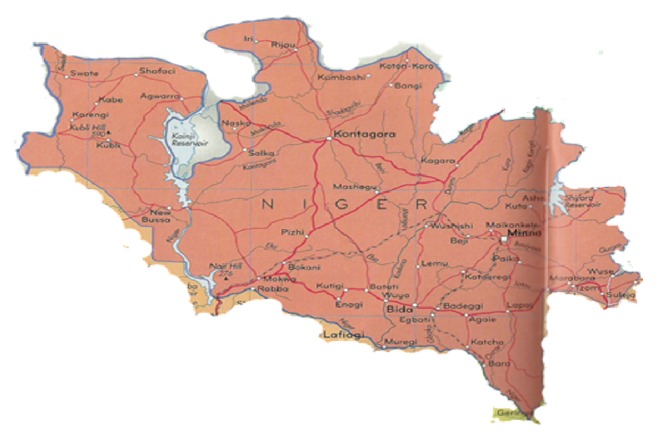
Map of Niger State, Nigeria. Source: Zaccheus Onumba Dibiaezue Memorial Libraries [21].

*Background*. Niger State (see Figure 1) is a state in the North Central Nigeria, covering a total land mass of 76,363 Km^2^ (about 10% of Nigeria's total land mass) and possessing an estimated population of 4,372,030 people (2,215,824 males and 2,156,206 females) [1]. It is the largest among the 36 states and the Federal Capital territory (FCT) of Nigeria and comprises 25 local government areas (LGAs).

Niger State has been identified as one of the three best states in Nigeria for malaria control [2]. The state was recognized by The Global Fund (GF) in 2012 for malaria Round 8 implementation efforts. It is one of the states where the Support for National Malaria Programme (SuNMAP), the Department for International Development Fund (DFID), and The Global Fund AIDS, Tuberculosis, and Malaria (GFATM) Round 8 are all implementing malaria project interventions [2].

The Association for Reproductive and Family Health (ARFH), a subrecipient to National Malaria Control Programme (NMCP) on GF Round 8 malaria in Niger State, was saddled with the responsibilities such as malaria case management through supply and tracking rapid diagnostic tests (RDTs) and artemisinin combination therapies (ACTs), advocacy communication, and social mobilization and health system strengthening through capacity building and monitoring and evaluation [3]. In the course of implementing the project, there were several instances in which patients demanded ACT without confirmatory tests or with negative RDT test results. Inequities in commodity procurement and distribution were also reported [4]. These irregularities provided the motivation for the current study.

According to WHO's recommendation, in malaria-endemic countries like Nigeria, everyone with suspected malaria should be diagnosed with RDTs or microscopy, and those testing positive should be treated with ACTs [5, 6]. On this basis, Nigeria adopted the requirement for the use of parasitological tests as one of the key interventions in improving malaria diagnosis and treatment [7, 8].

Prior to WHO's recommendation, malaria treatment in Niger State was based on clinical diagnosis (relying on signs and symptoms) instead of laboratory tests, according to the Niger State Health Management Information System (HMIS) database [9]. This finding is consistent with the results of a 2009 study in Enugu State, Nigeria, that showed over 50 percent presumptive treatment of malaria [10]. Due to the habitual practice of clinical diagnosis and symptomatic treatment of patients, health workers' capacity to conduct parasitological diagnosis fell below standard [11]. Symptomatic clinical diagnosis solely is not dependable, and difficulties with microscopy (both human and technical requirements) necessitated alternatives such as RDTs, which have been found to be eminently suitable [12].

The aforementioned study in Enugu State, which was conducted in a setting similar to Niger State, showed that the use of RDTs significantly reduced antimalaria drug prescriptions and was more cost effective when compared to other diagnostic methods for malaria in endemic areas [10]. Even in low transmission regions, RDTs have proven to be effective [13]. Studies conducted in Tanzania [14] and Ghana [15] showed that RDTs utilization improved correct treatment more than microscopy (microscopy led to excessive ACT prescription). Similarly, improvement was recorded in Uganda, where 39 percent reduction in ACT prescription occurred due to introduction of RDTs [16]. A study in Zanzibar [17] also showed that RDTs use decreased ACT prescription.

Access to malaria confirmatory tests is seriously underutilized in more than half of endemic African nations: at least 80 percent of malaria treatments are administered without diagnostic testing [18, 19]. RDTs are difficult to obtain, and supplies of ACTs are more than twice those of RDTs, making overuse of ACTs likely in both public and private sectors. Prices of ACTs are far beyond the previously used monotherapies, so investment in RDTs will reduce costs [20].

*Methodology*. In order to achieve the set objectives, the current study consists primarily of literature review, supported with data from the Niger State, HMIS data for 375 public health facilities (January–October 2013), and analyzed within a conceptual framework for assessing access to health services (as described below).

*Literature Review*. Peer-reviewed literature was obtained using a keyword search of databases (VU library and PubMed) and the use of Google Scholar. Grey literature was obtained from searches of institutional websites such as SuNMAP, ARFH, Niger State Government, Nigeria National Malaria Control Programme, and WHO.

The key search terms used singly and in combination included Niger State, Nigeria, Sub-Saharan Africa, rapid diagnostic tests, RDTs, artemisinin combination therapy, ACT, acceptance, barriers, availability, willingness, perception, stock-out, guidelines, malaria, and HMIS.

*Data Review*. The Global Fund commenced RDTs rollout in Niger State in 2012 with 250 public health facilities (HFs), while simultaneously providing malaria-specific HMIS training and documentation. In 2013 the number increased to 375 (15 per local government area (LGA)). In the current study, review of January to October HMIS 2013 data from these supported HFs were carried out. The choice of these HFs was based on completeness of data, timeliness of submission of reports, and compliance with the data quality assessment checklist. It is important to mention that a total of 1249 HFs (994 public and 255 private HFs), 1200 registered patent medicine stores, and 70 pharmacies were excluded from the study.

Analysis was based on key performance indicators recorded in the database, such as the numbers of persons in each of the following categories: presented with fever; presented with fever and tested using RDTs; presented with fever and tested using microscopy; clinically diagnosed for malaria; confirmed for uncomplicated malaria and treated with ACTs; and treated with ACTs on the basis of clinical diagnosis only [25]. Monthly totals for each indicator for the period under review were recorded. Several comparisons were made, including number of patients presented with fever versus parasitological confirmation with RDTs; quantity of ACTs versus quantity of RDTs supplied in 2013; number of patients with fever versus number of patients with positive test results; and patients with positive test results using RDTs versus number of patients treated at HFs.

**Figure 2:**
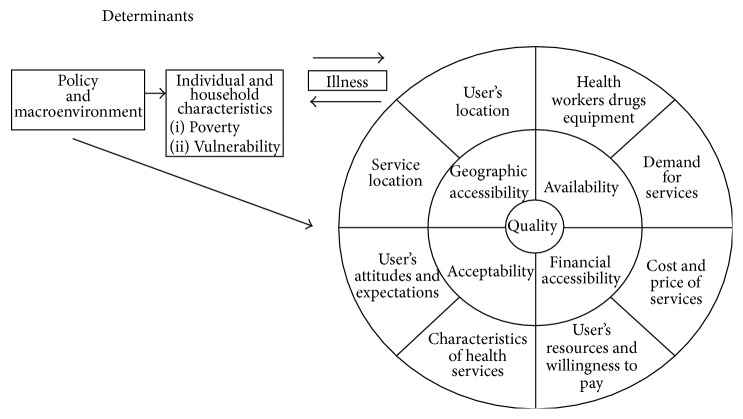
Conceptual framework for assessing access to health services [22].

*Conceptual Framework*. To effectively analyze access to malaria RDTs in Niger State, Nigeria, the conceptual framework of Peters et al. [22] was adapted. The framework was chosen because of its ability to address supply and demand elements of healthcare delivery in Sub-Saharan Africa. Thematic areas in the framework are shown in Figure 2. The current paper uses these areas in the framework to address accessibility barriers to RDTs as follows:

- policy and macroenvironment elements: (a) Nigeria national malaria diagnosis and treatment guidelines and (b) implementation of malaria diagnosis guidelines in Niger State,
- individual and household characteristics: (a) age and (b) educational attainment, poverty, and vulnerability,
- geographic accessibility: (a) service location and (b) user's location,
- financial accessibility: (a) cost and price of services and (b) user's resources and willingness to pay,
- availability: (a) health workers, drugs, and equipment and (b) demand for services,
- acceptability: (a) characteristics of health services and (b) user's attitudes and expectations,
- quality: quality of care, staff, treatment, capacity building, data, and commodity supplies.

## 2. Findings

### 2.1. Section Layout

This section presents results of the literature review, complemented by the analysis of Niger State secondary data, using Peters et al. [22] as a conceptual framework. The adapted conceptual framework ascribes utilization characteristics of RDTs to key elements of the framework, (1) policy and macroenvironment, (2) individual and household characteristics, (3) geographical accessibility, (4) financial accessibility, (5) availability, (6) acceptability, and (7) quality, which connects the previous four dimensions. In the following, we consider each of these elements in turn.

### 2.2. Policy and Macroenvironment

*Nigeria National Malaria Diagnosis and Treatment Guidelines as Contained in the Strategic Plan*. Nigeria's National Malaria Control Programme (NMCP) Strategic Plan for 2009–2013 consists of guidelines for diagnosis and treatment of malaria [26]. The plan expresses the goal of “timely and equitable access to malaria diagnosis and treatment by all sections of the population and as close to the home as possible” [26, page 24]. It is partly aligned with the 2010 edition of WHO's recommendation for parasitological confirmation of malaria by RDTs as one of the prioritized interventions in improving diagnosis and treatment.

At the national level, the expansion of access to RDTs use was aimed at increasing parasitological diagnosis to 40 percent in 2013 and 60 percent by 2014 [27]. These targets were unrealistic, as diagnosis using RDTs was limited and coverage was largely dependent on donors. The 2010 malaria indicator survey revealed that only 9.1 percent of children under five with fever in the north central region of Nigeria (including Niger State) had parasitological confirmation using RDTs [28].

A statewide rollout of RDTs in Oyo State, Nigeria, in 2012 was followed by increased ACT prescription rather than decrease. RDTs findings were not respected, as the ratio of ACT-treated patients to RDT-positive patients was consistently greater than 1 [29]. On the other hand, findings from a similar setting in Sudan showed that RDTs rollout was successful at the community through Home Management of Malaria (HMM) and led to improvement in treatment-seeking attitude of people [30]. A similar study in Uganda concluded that implementation of RDTs through community health workers (CHWs) required acceptance by the community, which was contingent on proper training, supervision, and logistical support of the CHWs [31].

*Implementation of Guidelines on Malaria Diagnosis in Niger State*. The 2009–2013 Strategic Plan for malaria in Niger State was implemented in collaboration with NMCP through GF Round 8. RDTs were first rolled out in 12 states in Nigeria, including Niger State, as a follow-up to WHO recommendation on parasitological diagnosis [26].

Translating the shift in guidelines into routine effective use of RDTs by service providers requires clear messages and guidelines that are adapted to local settings [32, 33]. Previously, in Niger State these requirements were not met. To improve this situation, capacity buildings were conducted at all HFs (375), and 400 Role Model Caregivers (RMCs) were supported by GF. Also, monthly review meetings, supportive supervision, and data collection were instituted [34].

### 2.3. Individual and Household Characteristics

“Individual and household characteristics have to do with socioeconomic position, vulnerability, and the health status. Poverty can be examined as a determinant of illness or health needs” [22]. 

*Age of Patient*. In Niger State, findings from analysis of state HMIS 2013 data showed that 40 percent of patients treated for malaria were children and approximately 21 percent of them were treated symptomatically through HMM [25]. Similarly, NDHS [36] showed that, for children aged 6–59 months with fever, 60.9 percent (*n* = 1, 268) of urban children and 41.2 percent (*n* = 2, 362) of rural children were treated for malaria presumptively during the two weeks prior to the survey. A qualitative study from Enugu State Nigeria showed that RDTs were rarely used for children due to their unavailability in consulting rooms, as well as doubts about their reliability [37]. It is common amongst caregivers and women to insist on prescription for their children, so that the use of RDTs for diagnosis is limited in younger age groups. In accordance with the 2013 Niger State Malaria Control Operational Plan (NMCOP), as long as RDTs are available at health facility almost all adults with fever were tested with RDTs [34].

*Educational Level, Poverty, and Vulnerability*. A study by NPC, NMCP, and ICF International [28] showed that educational attainment of caregivers and women correlates with their knowledge of how to care for their families and themselves. It follows that the higher the level of education, the greater the receptivity to healthcare products such as long-lasting insecticidal nets (LLINs) and RDTs.

Lack of education is positively correlated with poverty, and poverty restricts access to health services [22]. Currently, RDTs are free at supported health facilities in line with NMCOP 2013, but there are other costs that prevent people in the lowest wealth quintile from seeking malaria confirmatory tests, such as transportation to health facilities and lost income due to person-hours lost [38].

UNESCO [39] estimates literacy level for males and females in Niger State at 19.3 and 18.2 percent, respectively. Most of the population of Niger State consists of farmers with low financial capacity, according to NSMCP [9]. The currently low uptake of LLINs and RDTs corresponds with the low incomes and low level of educational attainment in the state.

### 2.4. Geographic Accessibility

Geographic accessibility is determined by “the physical distance or travel time from service delivery point to the user” [22, page 162]. 

**Figure 3:**
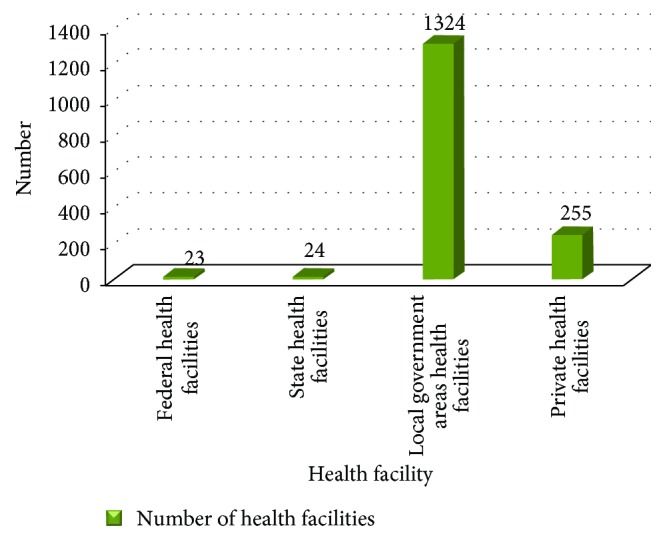
Number of health facilities by ownership. Source: NSMCP, 2013 [9].

*Service Location*. There are 1626 health facilities (HFs) in the state (see Figure 3). The health facilities are at 4 levels of ownership, comprising federal (23), state (24), local government (1324), and private (255). They are unevenly distributed compared with need: 40 percent of HFs are in urban areas that contain 30 percent of the population, while 60 percent of HFs are in rural areas, where 70 percent of the population resides [9].

Service locations include health facilities, Home Management of Malaria (HMM), and private sector (clinics, patent medicine stores (1200), and pharmacies (70)). The Society for Family Health (SFH) is implementing GFATM activities in the private sector via social marketing of ACTs. RDTs and ACTs are supplied by GFATM and SuNMAP to 375 public health facilities out of a total of 1624. The grant supports 15 HFs within each LGA. There are 16 RMCs affiliated to HFs based on proximity within the LGAs. The RMCs are responsible for presumptive treatment of malaria at the community level. They receive and replenish ACTs and report data to the HFs [40].

A critical factor for malaria diagnosis and case management is the location that people most often visit when they perceive they have malaria. More than 60 percent of clients purchase antimalarials in private health facilities and in most cases (95 percent) purchase monotherapy antimalaria drugs [41]. The 15 HFs per LGA are unevenly distributed and grossly inadequate for effective service coverage [9]. The poor coverage of HFs and services are due to low political will by the state government, which is evident from its underfunding of malaria case management.

According to the study of Awoleye [42], the terrain of the project location is crucial to effective implementation of public health programmes for malaria mitigation. For example, extensive seasonal flooding in Niger State inflicts hardship on programme implementers and limits access to healthcare by the users. 

*Users' Locations*. A study conducted in Kogi State, Nigeria, showed that only 18 percent of rural residents lived within 4 km of a public health center, compared to 40 percent that lived more than 10 km away from the nearest such facility. 62 percent of the residents close by sought medical treatment (and by proxy, malaria diagnostics and treatment) from government hospitals, compared to only 32 percent of distant residents [43].

In most rural communities, the first level of care for fever cases is home management, through either GFATM-funded HMM or patent medicine vendors. Caregivers usually visit health facilities only when there is no improvement. However, RDTs results will frequently be invalid or negative on patients that have already been treated with antimalarial drugs [9].

### 2.5. Financial Accessibility

Financial accessibility is defined as “the relationship between the price of services (in part affected by their costs) and the willingness and ability of users to pay for those services, as well as be protected from economic consequences of health costs” [22, page 162]. 

*User's Resources and Willingness to Pay*. There are several obstacles to access to public healthcare, among which is the cost of treatment [44]. The concept of “willingness to pay” (WTP) has been applied to healthcare products such as insecticide-treated nets in Nigeria [45]. A study conducted in Enugu State, Nigeria, by Uzochukwu et al. [46] showed that 51 percent of urban respondents were willing to commit resources to RDTs, compared to only 24.7 percent of rural respondents. Also, urban dwellers were ready to pay 235.49 naira, compared to 182.05 naira for rural residents. Since Niger State is made up of over 70 percent rural communities [47], this would indicate a relatively low level of WTP. A similar study in Uganda [48] showed that WTP for RDTs in drug shops was higher for customers with higher socioeconomic status, and WTP for RDTs was lower than that for ACTs. 

*Costs and Price of Services*. RDTs implementation in Niger State was donor-driven and was limited to selected public health facilities. The RDTs were free to patients. If donor funding stops, then access and affordability by patients to RDTs will be affected. Prices charged for RDTs in the private sector in Niger State are unknown. In Lagos Nigeria, RDTs prices range from $2.52 to $16.81 USD and average $5.88 USD, including the cost of charges on services such as diagnostic test but excluding consultation charges [49].

**Figure 4:**
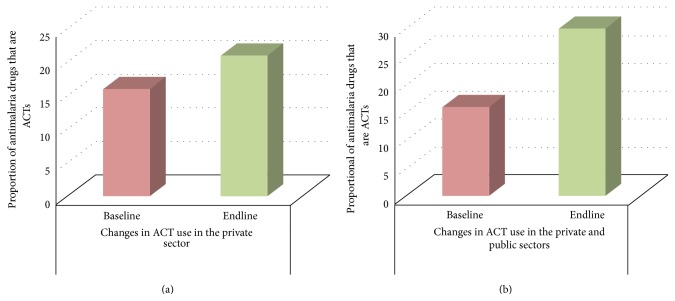
Changes in ACTs use in Nigeria 17 months before AMFm subsidy (baseline) and 15 months after subsidy (endline). Source: Morris et al. 2015 [23].

The Affordable Medicines Facility-Malaria (AMFm) was an intervention implemented at the private sector by the GFATM to increase access to ACTs and reduce the financial burden through subsidy [23].  Figure 4 shows changes in ACTs use in Nigeria before and after the introduction of subsidized ACTs in January 2011. The figure shows about 50 percent increase in ACTs following AMFm launch in both rural and urban areas. These results indicate that price is a key determinant of use of ACTs. Similar results should be expected with RDTs. However, there are relatively few private practitioners in the rural areas of Niger State, so the impact of private subsidies on such areas may be diminished.

### 2.6. Availability

Peters et al. characterize availability as “having the right type of care available to those who need it such as hours of operation and waiting times that meet demands of those who would use care, as well as having the appropriate type of service providers and materials” [22, page 162]. 

*Health Workers, Drug Equipment*. The “test, treat, and track” recommendation of WHO means that ACTs and RDTs are required and should be available in appropriate quantities [18, 19]. When RDTs were available in Niger State, there was a greater improvement in malaria case management at the primary health care level than was noticed in the secondary [9].

The number of patients that service providers have to attend to daily influences their performance and also determines waiting time of patients at health facilities. Both of these factors influence presumptive treatment.  Table 1 shows the available health workers in Niger State, who serve a population of greater than 4 million. There is definitely an extreme shortage of human resources for health in Niger State overall. The shortage of personnel with training on malaria control is even more extreme: ARFH trained 369 for RDTs (4 percent) personnel out of 9,083 that are directly involved in malaria control [34].

A study in Osun State in Nigeria emphasized that sparse access to health facilities produces a serious impact on sustainable growth through reduction of the number of workers' productive man hours [38]. Poor health facility accessibility in Enugu State Nigeria has contributed to self-diagnosis and medication by people in the lowest wealth quintile, and some turn to patent medicine vendors for treatment [50].

Due to poor working conditions in the public service and societal expectations, health workers in Lagos State, Nigeria, often seek opportunities abroad, leading to attrition of skilled labour in health [51]. This “brain drain” has not affected the public service of Niger State, especially because most cases of uncomplicated malaria are handled by lower-level health workers that are unlikely to resign from their appointments.

**Figure 5:**
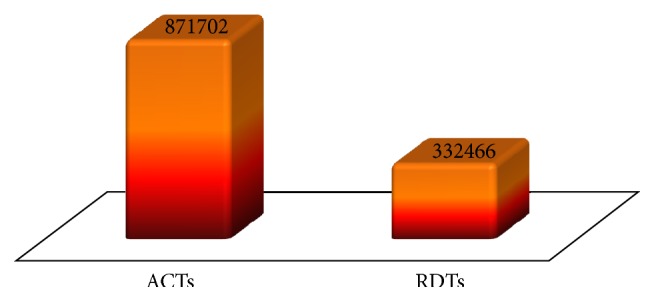
Quantity of ACTs and RDTs supplied to Niger State by SuNMAP and NMCP in 2013. Source: NSMCP, 2013 [9].

Figure 5 shows that roughly three times as many ACTs as RDTs were supplied by NMCP and SuNMAP in 2013. This is a typical case in which far more ACTs than RDTs are supplied to the service delivery points. In 2010, the ratio of RDTs to ACTs supplied by Global Fund was approximately 1 : 3. The Presidential Malaria Initiatives (PMI) also procured RDTs to ACTs in the ratio of 1 : 3 [27]. Ideally more RDTs than ACTs should be supplied, in order to allow for wastage and invalid results.

**Figure 6:**
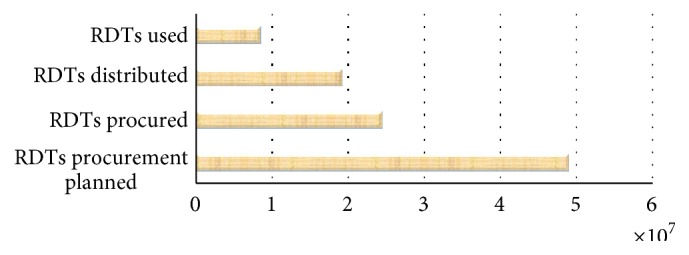
Programmatic results reported by GFATM malaria grant recipient on planning, distribution, and use of RDTs in 2010. Source: Zhao et al. 2012 [24].

Figure 6 shows the total GFATM RDTs planned (48,971,131), quantity procured (24,511,732), quantity distributed (19,219,064), and RDTs used (8,523,366) in 2010 for all African recipient countries [24]. Less than half of all RDTs distributed were actually used. These figures are for 2010, but Figure 5 shows that accessibility to RDTs remained a concern in 2013.

A study conducted in south-east Nigeria prior to the release of WHO's guideline showed that 0 percent of nonhospital treatment providers and 6 percent of hospitals used RDTs [52]. The study showed that providers' level of knowledge was poor: only 34.5 percent (resp., 73.5 percent) of nonhospital (resp., hospital) providers said that microscopy could be used to diagnose malaria. A 2012 study on the availability of RDTs in private facilities (clinics, pharmacies, private outlets, and laboratories) showed that only five out of one hundred and twenty (4.2 percent) such facilities in Lagos, Nigeria, had RDTs in stock [49].

**Figure 7:**
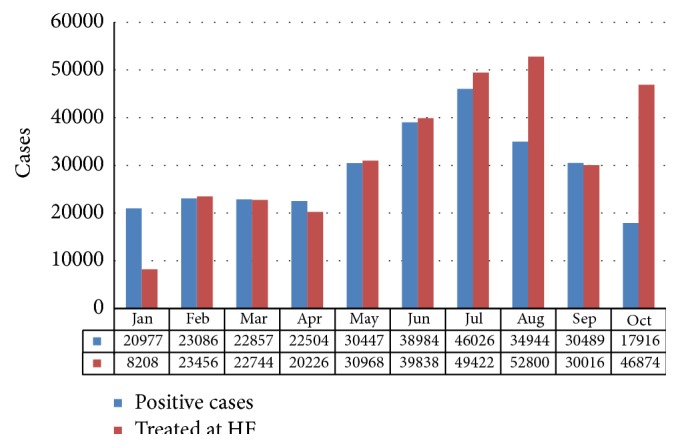
Number of patients with fever versus parasitological confirmation of malaria with RDTs, Niger State, January to October 2013 (HMIS data). Source: NSMCP, 2013 [9].

According to finding from HMIS, in the 375 Niger State-supported health facilities with RDTs, 77 percent of patients with fever received parasitological confirmation with RDTs [9, 25]. This may be due to poor access to RDTs as shown in Figure 7; not all patients that presented fever were tested.

Irregular and disproportionate supply of RDTs and ACTs in publically supported healthcare facilities is a major constraint in the implementation of RDT and quality of care in Niger State [53].  Table 2 shows an example from 2012.

In the first quarter of 2012, there were no RDTs in any public health facilities, even after ACTs were replenished in March. The Niger State stock supply pattern aligned with the findings of Zhao et al. [24] that rollout of RDTs always lags behind rollout of ACTs. ACTs and RDTs are supplied to health facility on a quarterly basis, typically in the ratio of 3 : 1 (as shown in Figure 5). In a health facility with high patient flow, it takes RDTs only two weeks to be out of stock. In most facilities, RDTs are out of stock by the third week of every month [53].

According to the supportive supervision report of ARFH [53], stockout of HMIS tools (data collection forms) constitutes accessibility constraint to knowing actual number of services provided and people reached. HMIS tools' availability is dependent on supplies from NMCP and donors [34].

*Demand for Services*. Pregnant women (PW) with confirmed malaria demonstrated demand for ACTs. However, only PW in the second and third trimesters could be issued ACTs, in line with Mens' [54] recommendation on malaria treatment for PW. HMM is a strategy for prompt treatment of children under five, but findings showed that adults also demanded treatment from RMCs [53]. There is no data available on demand for RDTs, but relevant information is covered in the following section on users' attitudes and expectations.

### 2.7. Acceptability

According to Peters et al. [22, page 162], acceptability is defined as “the match between how responsive health service providers are to the social and cultural expectations of individual users and communities.” 

*Users' Attitudes and Expectations*. No qualitative study on malaria diagnosis has been conducted in Niger State. A study in a similar situation in Enugu State, Nigeria, found that severe fever in children prompts their caregivers and parents to pressure health workers to treat children presumptively [37]. It is always difficult for women to wait for test results when their children exhibit serious symptoms such as vomiting and inability to eat food.

Patients often expect treatment based on symptoms alone, because of their belief that symptoms as shivering, excessive body temperature, diarrhea, and vomiting are sufficient to prove malaria [31]. Because of the cost and inconvenience involved in coming to the hospital, patients are unwilling to leave the hospital without some type of medication [37].

A study in Ghana showed that the presence of mosquitoes in patients' homes or environment influences their health-seeking behaviour. When they are feverish, they quickly conclude that mosquito bites were responsible and ask physician to prescribe antimalaria medication without further confirmation [55].

As far as patients' specific perceptions of RDTs, no study has been conducted in Niger State. However, studies elsewhere in Africa have shown that, especially in the rural areas, myths influence users' behaviour and practices. A study in Uganda found that some users were afraid that the drawing of blood from them or their children might cause HIV infections or be used for HIV testing. Some expressed anxiety that the blood could get into an enemy's hands and be used for witchcraft [31].

One author's (OJA) personal experience from supervisory visits to HFs in Niger State revealed that patients and caregivers expect that when the service provider conducts RDTs, then ACT should be available for treatment in case results are positive. However, in Niger State, at times RDTs were available while ACT was out of stock, or vice versa. When these patients' expectations were not met, it influenced subsequent acceptance of tests because of uncertainty whether treatment would be given.

*Characteristics of Health Services*. An important factor in user satisfaction with health services is preconsultation waiting times. A study of ophthalmological patients in southern Nigeria showed that long wait time was a major cause of patient dissatisfaction. Causes for long waits included nonadherence to appointment times, patients not seen in order of arrival, and lack of modern equipment that could shorten consultation time [56]. A study conducted in Kano State, Nigeria (a setting similar to Niger State), on patients' satisfaction with healthcare services, showed that 70 percent of respondents expressed satisfaction with health workers' behaviour, while 30 percent expressed dissatisfaction with long waiting times [57].

Health workers' adherence to guidelines reflects their responsiveness to users' expectations. In Kenya, lower-level healthcare providers such as nursing aides actually adhered to guidelines more closely compared to the higher-level staff such as clinical officers and nurses. Possible reasons for this included socialization effects (higher-level staff coming to view their own clinical judgment as superior to guidelines) and increased susceptibility to inappropriately prescribe second-line drugs [58]. In Niger State, 2013 programmatic report showed that irrespective of staff cadre, willingness and ability to adhere to guidelines were not the constraint, but rather availability of guidelines and RDTs [53].

Poor attitude among health workers may pose a major obstacle to acceptance of RDTs results by caregivers [55]. Additionally, rather than following treatment guidelines based on RDTs results, health workers may tend to follow “mindlines” that are shaped by poor diagnostic support, pressure from peers, and perceptions of patient preferences and from training emphasis of malaria as the most important disease [59].

### 2.8. Quality

Peters et al. [22, page 162] state that “quality of care is at the center of the circle of all four dimensions of access to health services, because it is an important component of each dimension and is ultimately related to the technical ability of health services to affect people's health.”

In the following, we present HMIS data that quantifies the quality of care of malaria patients at the 375 supported HFs in Niger State from January to October 2013.

Figure 7 shows the level of RDT testing on patients with fever. The rise in fever cases from May through October reflects the seasonal malaria pattern of Niger State. The drop in testing rate from August to October was due to depletion of RDTs stock [9]. The figure shows that not all patients with fever that attended received tests, although this is contrary to WHO diagnosis and treatment guidelines and is a serious quality issue for malaria case management.

**Figure 8:**
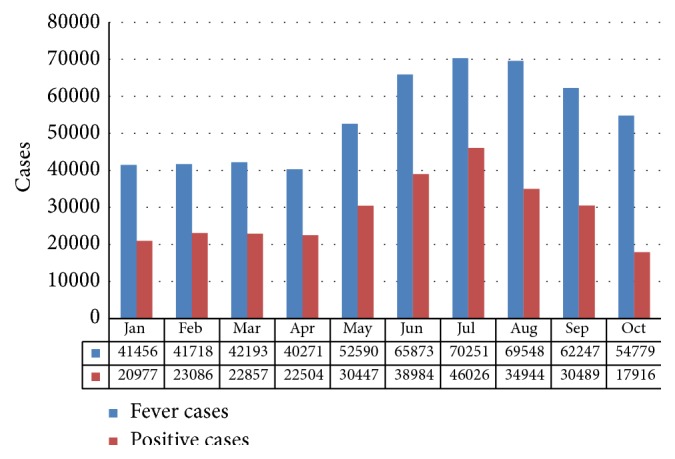
Number of patients at supported HFs with fever compared to number with positive test results, Niger State, January to October. Source: NSMCP, 2013 [9].

Figure 8 shows the number of patients with fever cases as against number confirmed in the 375 HFs that had RDTs available. RDTs identified a significant proportion of fever cases that did not require malaria treatment, especially during the first seven months of the period before RDTs stock became depleted. Altogether, 53 percent of fever cases tested positive with RDTs (but it should be noted that the percentage would have been higher if the RDTs stockout had not occurred).

**Figure 9:**
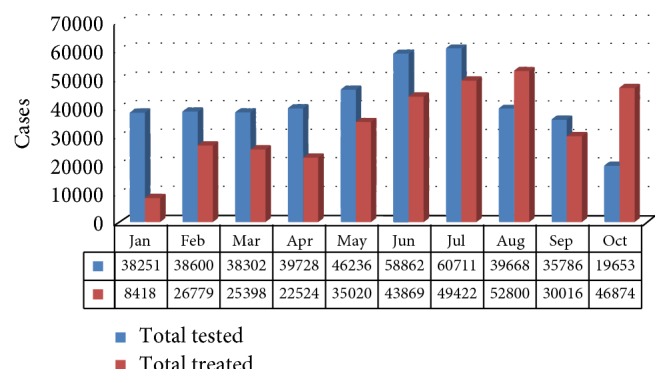
Total patients tested with RDTs as against total patients treated at health facility, Niger State, January to October 2013 HMIS. Source: NSMCP, 2013 [9].

Figure 9 shows that, in the months of August and October, the number of patients treated was greater than the number of patients that tested positive. This evidences presumptive treatment and at the same time reflects the depletion of RDTs in the latter part of the year.

**Figure 10:**
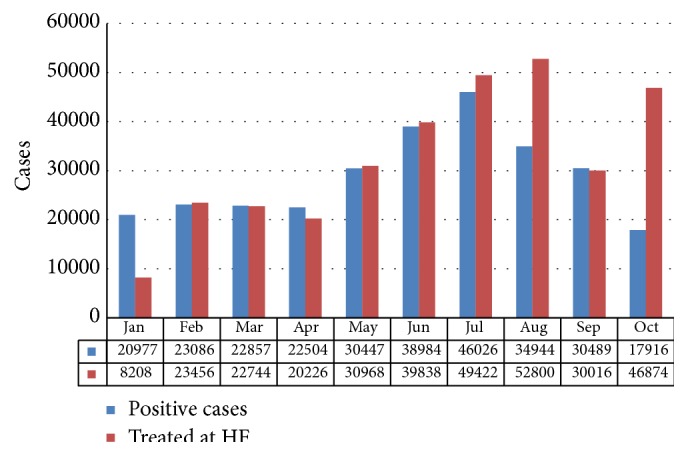
Patients tested positive using RDTs as against number of patients treated at supported health facilities in Niger State, January to October 2013 (HMIS data). Source: NSMCP, 2013 [9].

Figure 10 shows that, in the early part of the year, the ratio of treated patients to RDTs-positive patients was typically close to one. This would indicate that guidelines for treatment and diagnosis were followed rather closely, and ACT prescription was reduced as a result of the use of RDTs. These findings agree with those of Uzochukwu et al. [10] in Enugu State but differ from those of Ige and Ayandipo [29] in Oyo State that showed increased ACT prescription rates after RDTs rollout. However, the stock outages later in the year made it impossible to continue to follow guidelines, and ACT prescription rates increased.

To improve the quality of data, the state in collaboration with implementing partners (IPs) conducted training at all supported HFs on malaria case management, logistics, and Health Management Information System (HMIS) tools. This training helped service providers to understand indicators such as persons presenting with fever and tested using RDTs and persons tested positive for malaria using RDTs. In addition to the training, HMIS data-capturing tools were also procured: in order to facilitate reporting on services such as diagnosis and treatment, nearly one million malaria reporting (HMIS) and Logistic Management Information System (LMIS) tools were produced and distributed by the state government and implementing partners [34]. Within the private sector, SFH also built capacity of personnel responsible for data management [34].

Accessibility to RDTs from the service provider's perspective is subject to knowledge and availability of the guidelines at the point of use. It was observed by the national monitoring and evaluation team that treatment guidelines were frequently unavailable at the service delivery points: for example, in some cases, the guidelines were locked up or were kept at home by the head of the HF. In addition, there was no provision for translation of the guidelines from English into local languages, which may be a significant obstacle for some lower-level providers and for private healthcare [34].

To improve quality of case management, supportive supervision was initiated and conducted on monthly or quarterly bases by ARFH, SuNMAP, NMCP, and the state malaria control team. The LGA routinely supported health facilities, while the state team visited LGA and HFs monthly. NMCP and IPs visited the state and HFs on a quarterly basis [34].

Another factor that affected the quality of healthcare delivery was the inability of the Niger State malaria control program and partners to quantify accurately the actual commodity required by the state, which resulted from inconsistent consumption data. Commodity management (including tracking of ACTs and RDTs) and appropriate usage of bin card, request, and requisition forms were not adequately handled by the peripherals HFs [34].

The long time interval between training of health workers for RDTs and actual implementation may have had an adverse effect on the quality of testing. In Niger State, RDTs training was conducted in July 2011 while implementation commenced in June 2012 [53].

## 3. Discussion

In this section, we discuss best practices that enhance the use of RDTs.

*Using Community Health Workers (CHWs) to Implement RDTs.* In a study conducted from 2007 to 2008 in Livingstone District of Southern Province, Zambia, by Counihan et al. [60], community health workers (CHWs) received a half-day training on RDT use and interpretation and were then sent out with job aids, supplies of RDTs, and paracetamol to treat RDT-negative patients. Follow-up visits were paid to CHWs after 3, 6, and 12 months. Photographs of RDTs with positive, negative, and invalid test results were used to determine whether CHWs could identify results accurately. Findings showed that CHWs correctly identified results at 3rd month (87.7 percent) and at 12th month (100 percent). Also, interpretation of faint positive result drops to 76.7 percent at 12th months. The study concluded that training, supplies (RDTs, job aids, and consumables), and supervision can increase correct use of RDTs [60].

*Regular Supply of ACTs and RDTs*. In a qualitative study conducted in Ghana [61], one health worker expressed that patients rated her health facility higher than others due to presence of RDTs and ACTs, which improved patients' acceptance of RDT results and their confidence in the judgment of health workers on patients' conditions. Another health worker in the same study said that the availability of RDTs makes patients think the health facility has been upgraded with new technology, thereby increasing patient turnout for services. The study further explains how only availability of commodity will not sustain adherence but should be combined with effective communication and respect for patient.

Another study in Ghana showed that patients perceived accessibility of RDT kits as an advancement in malaria case management with regard to diagnosis and treatment. In order to achieve and sustain this confidence, a regular supply of ACTs and RDTs is required [55].

*Stock Monitoring.* A study in South Africa highlighted the following factors as having a positive influence on the appropriate use of RDTs: routine stock monitoring such as using stock cards (bin cards, request, and requisition forms) and/or electronic means such as logistic management information system (LMIS) and air-conditioning and monitoring of commodity room temperature with thermometers and regularly updated temperature charts [62].

*Education.* A study conducted in Southeast Nigeria showed that willingness to pay for RDTs increased with education of healthcare users, so that urban patients (who tend to be better educated) were more willing to pay for RDTs more than rural patients [46]. This finding aligns with NDHS 2013, which showed that increase in educational status of women correlates with increased antenatal care by skilled providers and health facility delivery [36]. Although RDTs and ACTs are free in Niger State, increased knowledge of RDTs among the population will facilitate accessibility [53].

*Social Marketing Increases RDTs Utilization.* A study conducted in Uganda from March 2011 to April 2012 demonstrated how a social marketing strategy can be used to increase RDTs utilization. The study took place in 67 villages and involved 92 registered shops. The identified shop owners were trained for RDTs administration and were also tasked with supplying data such as RDTs sales, numbers of positive and negative patients, and fees charged per patient. Monitoring checklists were furnished to shop owners to ensure that RDTs were administered appropriately, sharps and other wastes were handled appropriately, and RDTs were properly integrated in their sales. Following training, each shop owner was equipped with 40 RDTs, sharps disposal box, and free gloves for every RDT purchased. They were also connected with an RDTs wholesaler who subsequently supplied them with RDTs. The result showed that over 2,200 RDTs were sold monthly by trained shop owners over the 6-month period from July to December 2012 [63].

## 4. Conclusions

With support from NMCP, donors, and implementing agencies, Niger State has made significant progress in malaria control. However, to have the state free of malaria by year 2020 as enshrined in the state governor's “Niger State Vision 3:2020” [64], concerted efforts and commitment will be required by the state government. Although parasitological confirmation (using RDTs) of malaria has been officially adopted by Niger State, after almost half a decade the Niger State government still has not procured RDTs. Although the state government does procure ACTs, its supply is like a drop in the ocean.

Reliable availability of RDTs and ACTs also impacts acceptance. Commodity stock outages are a common occurrence at health facility supported due to irregular supply. Consumption rate per facility has not been established, and stock management is weak at the peripheral level due to poor adherence to logistic management information systems. When ACTs are out of stock, patients see no reason for testing without treatment.

On the part of donors, it is necessary to find out if they understand the programmatic implications of irregular and disproportionate supply of ACTs and RDTs. There may be a need for a formal memorandum of understanding between the state government and donors.

There is a general acceptance among the population of finger pricks for RDTs. However, acceptance of the result is an issue, especially when fever is severe.

Accessibility to RDTs result is motivated by perception of both patients and health care workers. When patient perceived that his/her fever is due to previous mosquito bites, a negative RDT result will not be sufficient to convince the patient. Also, to some healthcare providers, patient symptoms outweigh a negative RDT result.

The profit motive of registered private pharmacies (70) and patent medicine vendors (1200) in the state is a force that must be taken into consideration. With them, antimalaria drugs are rarely out of stock, and they are often the patient's first point of contact even before visiting a health facility. Although SFH is implementing GFATM activities at the private sector, still RDTs have not been integrated. This is a major barrier to RDTs implementation in Niger State, as well as other African settings.

Investment in RDTs by the state government will be a worthwhile venture, as it has been shown that a large fraction of fever cases presented do not require ACTs.

Supply in terms of personnel, commodities, and available health facilities is definitely less than demand for healthcare services including RDTs.

Malaria case management data are often from public health facilities. Much activity within the private sector is not captured; the few instances reported are donor funded (GFATM/SFH/SuMAP). Private sector actors that are not supported or recognized will not see the need to report data to the state government. Niger State can do simple things such as providing letters of recognition to private health facilities (PMVs and pharmacies), inviting them to stakeholders meetings, capacity building, and distribution of HMIS tools. These will motivate them to report on their activities.

## 5. Recommendations

- Health workers from nondonor-funded HFs should be trained on diagnosis using RDTs and be supplied with diagnosis and treatment guidelines.
- Expand access to malaria RDTs to HMM through awareness campaigns to the community. This should include education and training of RMCs as well as supplying them with RDTs.
- To effectively accommodate health workers at lower levels such as health post and RMCs, the guidelines should be translated into local languages for easy understanding.
- Establish consumption data on RDTs and ACTs that will inform quantity of commodity required at each level of healthcare and amount required for procurement.
- Invest in the procurement and proportionate distribution of RDTs to HFs (especially nonfunded HFs) and provide donor-funded RDTs during stock outages.
- The state government in collaboration with donors should support capacity building for private HFs' malaria diagnosis using RDTs and cascade RDTs' use through social marketing strategies in the private sector.
- Use existing platform (media, health systems, community-based organizations, etc.) to increase awareness and importance of RDTs in the case management of malaria.
- Perform continuous supportive supervision, such as visit to health facilities, phone calls, and institutionalized feedback mechanism to increase data quality and malaria case management. Quality data will support forecasting of RDTs and ACTs usage.
- Increase information, education, and communication messages to the general public (especially caregivers/women) on prompt response to childhood fever.
- A sustainability plan should be put in place, should the donors decide to withdraw or reduce support to the state.
- Procurement and distribution of RDTs should accompany ACTs and should be in a proportion that will allow invalid results, breakages, and retesting using RDTs.
- Donors supporting Niger State should be reminded of WHO and NMCP's guidelines on diagnosis and treatment, so that their commodities should be supplied in proportionate quantity.

## Figures and Tables

**Table 1:** Staff strength in Niger State.

Category	Total in the state
Doctors	234 (public = 167, private = 67)
Nurses/midwives	2037
Lab scientists	128
Lab technicians	43
Pharmacists	130 (public = 49, private = 81)
Pharmacy technicians	41
Trained role model caregivers (RMCs)	400
Community health officers (CHOs)	169
Community health extension workers (CHEWS)	2779
Environmental health officers	179
Social welfare workers	214
Registered patent medicine vendors (PMVs)	1200

Source: NMCOP, 2013 [34].

**Table 2:** Summary of First Quarter 2012 Stock Report.

Month	Percentage of public health facilities stocked out of ACTs	Percentage of public health facilities stocked out of RDTs
January	76%	100%
February	94%	100%
March	0%	100%

Source: ARFH, 2012 [35].
